# Association between Plasma Interleukin-27 Levels and Cardiovascular Events in Patients Undergoing Coronary Angiography

**DOI:** 10.3390/jcdd11050139

**Published:** 2024-04-30

**Authors:** Emi Saita, Yoshimi Kishimoto, Reiko Ohmori, Kazuo Kondo, Yukihiko Momiyama

**Affiliations:** 1 Research Institute of Environmental Medicine, Nagoya University, Nagoya 464-8601, Japan; 2 Department of Food Science and Human Nutrition, Faculty of Agriculture, Setsunan University, Osaka 573-0101, Japan; 3 Faculty of Regional Design, Utsunomiya University, Utusnomiiya 321-8505, Japan; 4 Faculty of Human Life and Environmental Sciences, Ochanomizu University, Tokyo 112-8610, Japan; 5 Department of Cardiology, NHO Tokyo Medical Center, Tokyo 152-8902, Japan

**Keywords:** atherosclerosis, biomarkers, cardiovascular events, coronary heart disease, IL-27

## Abstract

Atherosclerotic disease, including coronary heart disease (CHD), is one of the chronic inflammatory conditions, and an imbalance between pro-inflammatory and anti-inflammatory cytokines plays a role in the process of atherosclerosis. Interleukin (IL)-27, one of the IL-12 family members, is recognized to play a dual role in regulating immune responses with both pro-inflammatory and anti-inflammatory properties. IL-27 is secreted from monocytes, T cells, and endothelial cells, and its expression is upregulated in atherosclerotic plaques. We previously reported that no significant difference was observed in plasma IL-27 levels between patients with stable CHD and those without it. However, the prognostic value of IL-27 levels has not been fully elucidated. We studied the relation of plasma IL-27 levels to cardiovascular events in 402 patients undergoing elective coronary angiography for suspected CHD. We defined cardiovascular events as cardiovascular death, myocardial infarction, unstable angina, stroke, or coronary revascularization. Of the 402 study patients, CHD was present in 209 (52%) patients. Plasma IL-27 levels were not markedly different between patients with CHD and those without it (median 0.23 vs. 0.23 ng/mL). During a follow-up of 7.6 ± 4.5 years, cardiovascular events were observed in 70 patients (17%). In comparison to the 332 patients with no event, the 70 patients who had cardiovascular events showed significantly higher IL-27 levels (median 0.29 vs. 0.22 ng/mL) and more frequently had an IL-27 level of >0.25 ng/mL (59% vs. 40%) (*p* < 0.01). The Kaplan–Meier analysis demonstrated a lower event-free survival rate in patients with an IL-27 level >0.25 ng/mL than in those with an IL-27 level ≤0.25 ng/mL (*p* < 0.02). The multivariate Cox proportional hazards regression analysis showed that IL-27 level (>0.25 ng/mL) was a significant predictor for cardiovascular events (hazard ratio: 1.82; 95%CI: 1.13–2.93, *p* < 0.02), independent of CHD. Thus, high IL-27 levels in plasma were related to an increased risk of further cardiovascular events in patients who underwent elective coronary angiography.

## 1. Introduction

Atherosclerotic diseases, including coronary heart disease (CHD), are chronic inflammatory conditions, and atherosclerotic plaques develop due to an imbalance between pro-inflammatory and anti-inflammatory cytokines [1]. The T-helper (Th) cells differentiate into the Th1 and Th2 cells. The Th1 cells have the pro-inflammatory effects of secreting cytokines like interferon (IFN)-γ and interleukin (IL)-2, whereas the Th2 cells induce anti-inflammatory responses via the secretion of IL-4, IL-10, and IL-13 cytokines. Furthermore, regulatory T (Treg) cells are a subtype of CD4^+^ T cells that regulate the effects of Th1 and Th2 cells. The pathogenesis of inflammation in atherosclerosis is attributable to the imbalance between pro-inflammatory Th1 and anti-inflammatory Th2 cytokines and impaired Treg responses [2,3].

In 2002, Pflanz et al. [4] first identified IL-27 as one of the IL-12 family members that is a heterodimeric cytokine consisting of the p28 subunit (an IL-6 and p35 homologue) and the Epstein–Barr virus-induced gene 3 (EBI3) subunit (an IL-12 p40 homologue that was originally discovered to be secreted from Epstein–Barr virus-transformed B cells). IL-27 is mainly secreted from monocytes, T cells, endothelial cells, and dendritic cells [2,4]. Moreover, IL-27 binds to the IL-27 receptor (IL-27R), consisting of a ligand-binding chain, the IL-27 Rα (WSX-1) subunit, which is unique for the IL-27 binding, and an additional signal-transducing chain, the gp130 subunit, which is shared with the IL-6 receptor [5,6]. IL-27R is expressed in various cells, like T cells, macrophages, dendritic cells, and endothelial cells [2,7]. Both IL-27 and IL-27R gene expression was demonstrated to be upregulated in atherosclerotic plaques [8]. In human endarterectomy specimens from the carotid artery, IL-27 expression was shown in vascular smooth muscle cells (SMCs), endothelial cells, and macrophages [9]. 

IL-27 is now recognized to play a dual role in regulating immune responses with pro-inflammatory and anti-inflammatory properties [5,10]. IL-27 was shown to promote naive T cell proliferation, induce Th1 differentiation and polarization, and increase IFN-γ production in naive CD4^+^ T cells [2,4]. IL-27 is a strong inducer of pro-inflammatory cytokines, like IL-1β, IL-6, and TNF-α, in monocytes [6,11]. IL-27 was also shown to promote LPS-induced IL-1β secretion in monocytes and macrophages [12]. Furthermore, IL-27 has an inhibitory effect on Th2 cell differentiation and cytokine production, like IL-5 and IL-13 [13]. On the other hand, IL-27 was reported to inhibit IL-2 production and promote IL-10 production by CD4^+^ T cells, thus suggesting anti-inflammatory properties. IL-27 also inhibits Th17 differentiation and IL-17 production [5,10,13]. Furthermore, IL-27 promotes the generation of IL-10-producing regulatory T cells [14]. However, the strong upregulation of IL-27 was shown on cultured aortic SMCs by TNF-α and INF-γ stimulation [9]. In monocyte-derived dendritic cells, oxidized LDL also upregulated IL-27 mRNA and protein expression [15]. IL-27 was reported to enhance TNF-α-mediated upregulation of adhesion molecules, such as ICAM-1 and VCAM-1, pro-inflammatory IL-6, and chemokines CCL5 and CXCL10 in cultured human coronary artery endothelial cells [16]. IL-27 also induced Th1 differentiation and upregulated ICAM-1 expression in naive CD4^+^ T cells [17]. These findings thus indicate that IL-27 would have a primarily promotive effect on atherosclerosis as well as inflammation.

Regarding blood IL-27 levels and atherosclerotic diseases, several studies reported high blood IL-27 levels in patients who had acute coronary syndrome (ACS), defined as acute myocardial infarction (MI) or unstable angina pectoris (UAP) [15,18,19]. Recently, Grufman et al. [20] evaluated plasma IL-27 levels and prognosis in ACS patients, and they showed that high IL-27 levels were related to recurrent MI and cardiovascular death. However, any association of blood IL-27 levels with cardiovascular events in patients who underwent elective coronary angiography or patients with stable CHD has not been fully elucidated. Notably, we previously measured plasma IL-27 levels in 147 patients with stable CHD and 97 without it and reported that no significant difference was observed in IL-27 levels between patients with stable CHD and those without it [21]. To elucidate the prognostic value of plasma IL-27 levels in patients who underwent elective coronary angiography for suspected CHD, the present study extended our previous study [21] by increasing the number of patients and then by following up for cardiovascular events.

## 2. Materials and Methods

### 2.1. Patient Population

In July 2008, we began to prospectively collect blood samples as well as clinical and angiographic data from patients who underwent coronary angiography for suspected CHD at NHO Tokyo Medical Center in Japan. Patients who had any history of percutaneous coronary intervention (PCI) or coronary artery bypass grafting (CABG), or patients who were on hemodialysis were not asked for their participation in our study. The institutional ethics committee had approved our study (Approval Number: R08-050 and R21-037). After taking written informed consent according to the Helsinki Declaration, blood sampling was performed in an overnight fasting state on the morning of the day when angiography was performed. Any patients who were admitted for ACS, defined as acute MI or class III UAP by Braunwald’s classification [22], or those who had any history of heart failure (HF), were excluded from the present study. Since serum IL-27 levels have been documented to be increased in patients with breast or lung cancers [23,24], patients who had any cancer were also excluded. In the present study, we assessed plasma IL-27 levels in 402 consecutive patients who underwent elective coronary angiography for suspected CHD and then were followed for a mean period of 7.8 ± 4.5 years for cardiovascular events. We defined hypertension as having blood pressures ≥140/90 mmHg and/or drug prescriptions; 243 patients (60%) were taking anti-hypertensive medication. We also defined hypercholesterolemia as having an LDL cholesterol level >140 mg/dL and/or drug prescriptions, and 146 patients (36%) were taking statin. Diabetes mellitus (DM) was defined as having a fasting plasma glucose level ≥126 mg/dL and/or drug prescriptions or insulin treatment, and 100 patients (25%) were found to have DM. We defined smoking as a history of 10 or more pack-years smoking, and 171 patients (43%) had such a smoking history.

### 2.2. The Measurements of IL-27 and C-Reactive Protein Levels in Plasma

Blood samples were collected into tubes with EDTA and centrifuged at 2000× *g* for 15 min at 4 °C. Plasma was frozen and stored at −80 °C until use. For the measurement of IL-27 levels, the enzyme-linked immunosorbent assay (ELISA) (LEGEND MAX™ Human IL-27 ELISA Kit; BioLegend, San Diego, CA, USA) was used. As previously reported [21], we assessed IL-27 levels at Ochanomizu University according to the manufacturer’s instructions. According to data from the manufacturer, the lowest detection limit of this kit was 0.01 ng/mL. The intra- and interassay coefficients of variation were found to be <6.0% and <5.5%. To measure high-sensitivity C-reactive protein (CRP) levels, a BNII nephelometer (Siemens Healthineers, Tokyo, Japan) was used.

### 2.3. Coronary Angiography at Baseline and Clinical Follow-Up

We performed angiography using the Philips Electronics angiogram system (Tokyo, Japan). CHD was defined as ≥1 coronary artery having >50% stenosis, and the severity of CHD was evaluated as the number of vessels having >50% stenosis. The stenosis severity in each segment by the CASS classification was assessed from a visual assessment and was classified into 5 grades (<25%, 26–50%, 51–75%, 76–90%, and >90% stenosis). All the angiograms were evaluated by a single cardiologist, who was blind to clinical and laboratory data. Left ventricular (LV) systolic function was evaluated as LV ejection fraction (LVEF) measured using echocardiography. For a mean period of 7.6 ± 4.5 years, all our patients were followed for cardiovascular events. As in our previous report [25], we defined cardiovascular events as cardiovascular death, MI, hospitalization for UAP or stroke, or the need for coronary revascularization, such as PCI and/or CABG. However, if PCI or CABG were scheduled and performed as a result of coronary angiography at baseline, they were judged not to be events. The patients’ outcomes were assessed by a review of their medical records. 

### 2.4. Statistical Analysis

We conducted all statistical analyses using the IBM SPSS version 29 software and defined statistical significance as a *p*-value < 0.05. Parametric and categorical parameters were presented as the mean ± SD and the number (%), respectively. As the measured CRP and IL-27 levels were judged to be nonparametric by the Shapiro–Wilk test, their results were represented as the median value and the interquartile range. For parametric, nonparametric, and categorical parameters, the unpaired *t*-test, Mann–Whitney U test, and chi-square test were used to assess any differences between two groups, respectively. The optimal cutoff point of IL-27 for cardiovascular events was found to be 0.25 ng/mL, where the Youden index of sensitivity + specificity − 1 is the maximum [26]. The event-free survival rates in patients with an IL-27 level of >0.25 ng/mL and those with IL-27 ≤0.25 ng/mL were compared using the Kaplan–Meier method with a log-rank test. As for the cut-off point of CRP, the previously shown cut-off point of 1.0 mg/L was used [27,28]. A multivariate Cox proportional hazards regression analysis was performed to find the independent predictors for cardiovascular events.

## 3. Results

Of the 402 patients, CHD (>50% stenosis) was observed in 209 (52%) patients, of whom PCI and CABG were performed in 111 and 39 patients, respectively, as a result of angiography at baseline. In comparison to the 193 patients without CHD, the 209 with CHD were significantly older, had a male predominance, and more frequently had hypertension, hypercholesterolemia, DM, and lower HDL cholesterol levels. Furthermore, plasma CRP levels were significantly higher in patients with CHD than in patients without CHD (median 0.80 vs. 0.51 mg/L, *p* < 0.005) (Table 1). There was no significant difference in plasma IL-27 levels between patients with CHD and patients without CHD (median 0.23 vs. 0.23 ng/mL, *p* = NS).

During the mean follow-up of 7.6 ± 4.5 years, cardiovascular events were observed in 70 (17%) patients (cardiovascular death, *n* = 20; MI, *n* = 5; UAP, *n* = 8; stroke, *n* = 12; coronary revascularization, *n* = 25). In comparison to the 332 patients with no event, the 70 patients with cardiovascular events had higher LDL cholesterol and lower HDL cholesterol levels (*p* < 0.05). Moreover, patients with cardiovascular events had a higher prevalence of CHD (80% vs. 46%) and a greater number of >50% stenotic coronary vessels (1.7 ± 1.1 vs. 0.8 ± 1.0) (*p* < 0.001) (Table 2). CRP levels were higher in patients with events than in those with no events (0.85 vs. 0.60 mg/L), but this difference did not reach statistical significance. Of note was that patients with events had significantly higher plasma IL-27 levels (0.29 vs. 0.22 ng/mL) and more often had an IL-27 level of >0.25 ng/mL (59% vs. 40%) than those with no event (*p* < 0.01). As a result, the sensitivity and specificity to predict cardiovascular events for the IL-27 level of >0.25 ng/mL were 59% and 60%, and the positive and negative predictive values were 24% and 87%, respectively.

**Figure 1:**
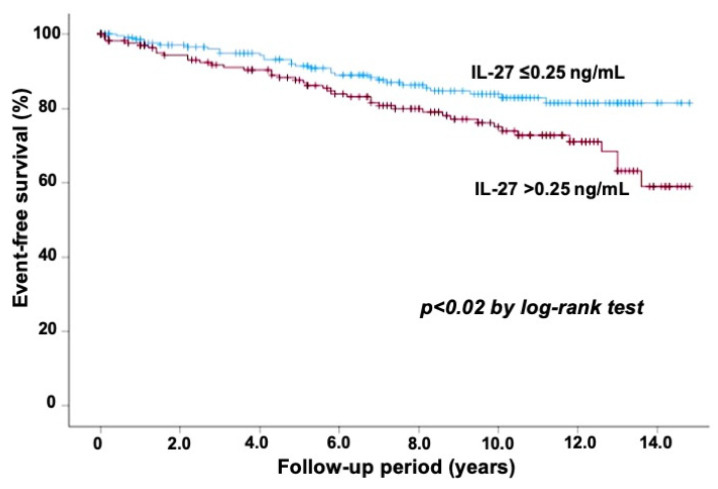
Event-free survival from cardiovascular events in 402 study patients. Kaplan–Meier analysis showed lower event-free survival in patients with an IL-27 level >0.25 ng/mL than in those with an IL-27 level ≤0.25 ng/mL (*p* < 0.02).

**Figure 2:**
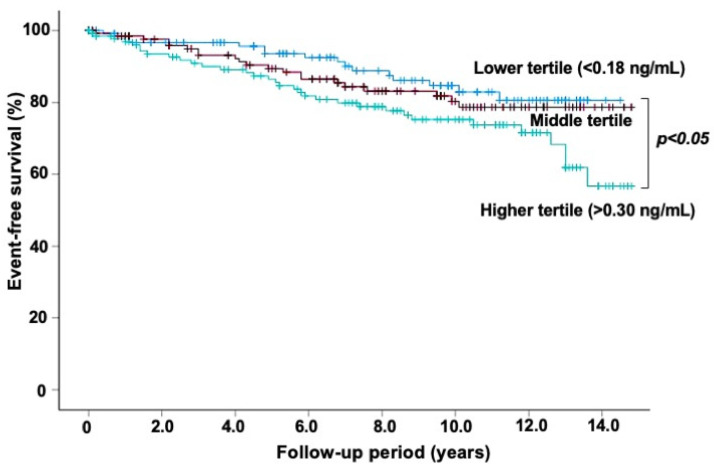
The event-free survival from cardiovascular events. The 402 patients were divided into tertiles according to IL-27 levels: lower (<0.18 ng/mL), middle (0.19–0.30 ng/mL), and higher (>0.30 ng/mL) tertiles. A Kaplan–Meier analysis showed lower event-free survival in patients in the higher tertile compared with those in the lower tertile (*p* < 0.05).

To elucidate the relation of IL-27 levels to cardiovascular events, the 402 patients were divided into two groups according to IL-27 levels (>0.25 and ≤0.25 ng/mL). The Kaplan–Meier analysis demonstrated lower event-free survival in patients with an IL-27 level >0.25 ng/mL than in those with IL-27 level ≤0.25 ng/mL (*p* < 0.02) (Figure 1). In a multivariate Cox proportional hazards regression analysis, IL-27 level and CHD were independent predictors of cardiovascular events, but CRP level was not. The hazard ratio (HR) of IL-27 >0.25 ng/mL for cardiovascular events was 1.82 (95%CI: 1.13–2.93, *p* < 0.02) (Table 3). To further clarify the relation between IL-27 levels and cardiovascular events, the 402 patients were divided into the tertiles of IL-27 levels: lower (<0.18 ng/mL), middle (0.19–0.30 ng/mL), and higher (>0.30 ng/mL) tertiles. Kaplan–Meier analysis showed significantly lower event-free survival in patients in the higher tertile than those in the lower tertile (*p* < 0.05) (Figure 2). In multivariate Cox proportional hazards analysis, compared with patients in the lower tertile, those in the middle and higher tertiles had HRs of 1.29 (95%CI = 0.66–2.51) and 1.97 (95%CI = 1.07–3.62, *p* < 0.05) for cardiovascular events, respectively.

## 4. Discussion

We studied the prognostic value of plasma IL-27 levels in 402 patients who underwent elective coronary angiography for suspected CHD. Plasma IL-27 levels were not markedly different between patients with stable CHD and those without it. Of note was that IL-27 levels were significantly higher in patients who developed cardiovascular events than in those with no event. High plasma IL-27 levels were related to an increased risk of further cardiovascular events, independent of CHD, CRP levels, and atherosclerotic risk factors. 

IL-27 is suggested to have dual effects on regulating the immune response with pro-inflammatory and anti-inflammatory properties [5,10]. However, the role of IL-27 in promoting or suppressing inflammation may vary within different diseases. IL-27 promotes inflammation in diseases such as crescentic glomerulonephritis, colitis, and systemic sclerosis, but IL-27 suppresses inflammation in diseases such as autoimmune arthritis, allergic asthma, and autoimmune encephalomyelitis [15]. Regarding the role of IL-27 in atherosclerosis, IL-27 enhanced the upregulation of adhesion molecules and pro-inflammatory cytokines in cultured endothelial cells [16]. IL-27 also promoted Th1 differentiation and upregulated ICAM-1 on CD4^+^ T cells [17]. These findings indicate that IL-27 may play a primarily promotive role in atherosclerosis and inflammation. In contrast, in animal models of atherosclerosis, recombinant IL-27 administration inhibited the progression of atherosclerosis in ApoE-deficient mice [29]. Ldlr^−/−^ mice transplanted with IL-27 receptor^−/−^ bone marrow showed larger atherosclerotic lesions [30]. IL-27-deficient mice also developed increased atherosclerosis with enhanced macrophage activation [7]. However, mice with IL-27R genetic ablation were reported to be protected against the development of aortic aneurysms [31]. Therefore, the effect of IL-27 on the process of atherosclerosis still remains a matter of debate.

As for blood IL-27 levels and atherosclerosis, plasma lL-27 levels were reported to be higher in 140 patients who had carotid artery stenosis compared with 19 healthy controls [8]. Ye et al. [32] also measured plasma IL-27 levels in 430 hypertensive patients and reported IL-27 levels to be associated with carotid atherosclerotic plaques. Several studies reported blood IL-27 levels in patients with ACS, which was defined as acute MI or UAP, to be high [15,18,19]. Moreover, Si et al. [33] measured serum IL-27 levels in 81 patients with Kawasaki disease and 90 healthy controls and showed IL-27 levels to be higher in patients with Kawasaki disease, especially in such patients with coronary arterial lesions, than in controls. Regarding blood IL-27 levels and CHD, Jin et al. [15] measured plasma IL-27 levels in 30 patients with stable CHD and 27 without CAD and showed IL-27 levels to be higher in patients with CHD than in those without CHD. In contrast, Lin et al. [18] reported no significant difference in plasma IL-27 levels between 43 patients with stable CHD and 47 without it. Although the present study extended our previous report [21] by increasing the number of patients (from 244 up to 402 patients), we found that plasma IL-27 levels were not markedly different between the 209 patients with stable CHD and the 193 without it. Of note was that plasma IL-27 levels were significantly higher in the 70 patients who developed cardiovascular events than in the 332 patients with no events. High IL-27 levels were related to an increased risk of cardiovascular events, independent of the presence of CHD. 

Regarding blood IL-27 levels and clinical outcome, Eric et al. [34] studied the association of serum IL-27 levels with in-hospital mortality in 151 critically ill patients with peritonitis, pancreatitis, or trauma who were admitted to the intensive care units, and they reported that IL-27 levels on admission were significantly higher in patients who died during hospital than in those who survived. Xu et al. [35] assessed serum IL-27 levels in 239 patients with community-acquired pneumonia and showed that higher IL-27 levels were related to an increased risk of vasoactive agent usage and a longer hospital stay. Recently, Grufman et al. [20] assessed plasma IL-27 levels in 524 patients with ACS and followed them up during the median follow-up of 2.2 years. The incidence of the combined end-point of MI and cardiovascular death was significantly higher in patients with IL-27 within the top two tertiles than in those with the lowest tertile, suggesting an association between high IL-27 levels and a worse prognosis in patients with ACS. Our present study, for the first time, reported that plasma IL-27 levels were significantly higher in patients with cardiovascular events than in those without such events among 402 patients undergoing elective coronary angiography for suspected CHD. High IL-27 levels were related to an increased risk of cardiovascular events independent of CHD, but CRP levels were not independent predictors of cardiovascular events. Our results indicate that high plasma IL-27 levels can be a biomarker for further cardiovascular events in patients who underwent elective coronary angiography.

Our study was associated with some limitations. First, our study population was relatively small (402 patients), and the number of patients who had cardiovascular events was especially small (70 patients). To clarify the prognostic value of IL-27 levels, further studies in a larger number of study patients will be needed. Second, we performed coronary angiography to evaluate the presence and severity of CHD. Angiography is unable to look at coronary artery plaques but only shows the lumen characteristics of the artery. Moreover, the severity of stenosis was not assessed by quantitative angiography or coronary fractional flow reserve; it was assessed only by the visual assessment of a single cardiologist, like in our previous study [21]. These may have affected our results. Third, our study population consisted of Japanese patients who underwent coronary angiography. Such patients were generally recognized to be a highly select population at high risk for CHD. Therefore, our results may not be applicable to general or other ethnic populations. Finally, we assessed plasma IL-27 levels only at baseline angiography and did not evaluate any changes in IL-27 levels during the period of follow-up, which may have affected outcomes. Furthermore, we did not assess any changes in medication for the treatment of CHD, which may have confounded our results.

## 5. Conclusions

The present study investigated the prognostic value of plasma IL-27 levels in patients who underwent elective coronary angiography for suspected CHD. Plasma IL-27 levels were not markedly different between patients with stable CHD and those without it. However, IL-27 levels were higher in patients with cardiovascular events than in those with no events. High IL-27 levels were found to be related to an increased risk of cardiovascular events, independent of CHD, CRP levels, and atherosclerotic risk factors. Our results indicate that high IL-27 levels in the blood can be a biomarker of further cardiovascular events in patients undergoing elective coronary angiography.

## Figures and Tables

**Table 1:** Clinical data and IL-27 levels in patients with CHD and those without CHD.

	All			
	(*n* = 402)	CHD (−) (*n* = 193)	(−) vs. (+)	CHD (+) (*n* = 209)
Age (years)	67 ± 11	65 ± 12	<0.001	69 ± 10
Sex (men)	276 (69%)	116 (60%)	<0.001	160 (77%)
Hypertension	285 (71%)	119 (62%)	<0.001	166 (79%)
Hypercholesterolemia	207 (51%)	80 (41%)	<0.001	127 (61%)
LDL cholesterol (mg/dL)	114 ± 31	111 ± 29	NS	116 ± 33
HDL cholesterol (mg/dL)	54 ± 15	58 ± 16	<0.001	51 ± 13
Statin	146 (36%)	48 (25%)	<0.001	98 (47%)
DM	100 (25%)	26 (13%)	<0.001	74 (35%)
Smokers	171 (43%)	68 (35%)	<0.01	103 (49%)
LV ejection fraction (%)	63 ± 10	64 ± 9	NS	62 ± 10
CRP levels (mg/L)	0.62 [0.30, 1.53]	0.51 [0.27, 1.24]	<0.005	0.80 [0.37, 1.74]
CRP level > 1.0 mg/L	145 (36%)	59 (31%)	<0.05	86 (41%)
IL-27 levels (ng/mL)	0.23 [0.15, 0.35]	0.23 [0.14, 0.34]	NS	0.23 [0.16, 0.35]
IL-27 level > 0.25 ng/mL	173 (43%)	80 (41%)	NS	93 (44%)

The data represent the mean value ± SD or the number (%), except for CRP and IL-27 levels, which represent the median value and interquartile range.

**Table 2:** Clinical data and IL-27 levels in patients with cardiovascular events and those with no event.

	All			
	(*n* = 402)	Event (−) (*n* = 332)	(−) vs. (+)	Event (+) (*n* = 70)
Age (years)	67 ± 11	66 ± 11	NS	69 ± 11
Sex (men)	276 (69%)	224 (67%)	NS	52 (74%)
Hypertension	285 (71%)	231 (70%)	NS	54 (77%)
Hypercholesterolemia	207 (51%)	169 (51%)	NS	38 (54%)
LDL cholesterol (mg/dL)	114 ± 31	112 ± 30	<0.05	121 ± 33
HDL cholesterol (mg/dL)	54 ± 15	55 ± 15	<0.02	50 ± 14
Statin	146 (36%)	117 (35%)	NS	29 (41%)
DM	100 (25%)	80 (24%)	NS	20 (29%)
Smokers	171 (43%)	139 (42%)	NS	32 (46%)
CHD	209 (52%)	153 (46%)	<0.001	56 (80%)
Number of stenotic vessels	1.0 ± 1.1	0.8 ± 1.0	<0.001	1.7 ± 1.1
LV ejection fraction (%)	63 ± 10	63 ± 9	NS	62 ± 12
CRP levels (mg/L)	0.62 [0.30, 1.53]	0.60 [0.29, 1.50]	NS	0.85 [0.43, 1.77]
CRP level > 1.0 mg/L	145 (36%)	114 (34%)	NS	31 (44%)
IL-27 levels (ng/mL)	0.23 [0.15, 0.35]	0.22 [0.15, 0.34]	<0.01	0.29 [0.19, 0.40]
IL-27 level > 0.25 ng/mL	173 (43%)	132 (40%)	<0.01	41 (59%)

The data represent the mean value ± SD or the number (%), except for CRP and IL-27 levels, which represent the median value and interquartile range.

**Table 3:** Independent factors for cardiovascular events in 402 patients.

	Hazard Ratio	(95% CI)	*p* Value
CHD	3.57	(1.98–6.41)	<0.001
IL-27 (>0.25 ng/mL)	1.82	(1.13–2.93)	<0.02

The dependent variable was cardiovascular events. Included were age, sex, hypertension, hypercholesterolemia, stain, smokers, DM, CHD, coronary revascularization at baseline, and LV ejection fraction, along with HDL-cholesterol (<40 mg/dL), CRP (>1.0 mg/L), and IL-27 (>0.25 ng/mL) levels.

## Data Availability

The data that support the findings of our study are available from the corresponding author on reasonable request.
